# Supplementary material for: Heme biosynthesis depends on previously unrecognized acquisition of iron-sulfur cofactors in human amino-levulinic acid dehydratase
Source: Nat Commun. 2020 Dec 9;11:6310. doi: 10.1038/s41467-020-20145-9 (PMC7725820; doi:10.1038/s41467-020-20145-9)
Supplement: Supplementary file 1 — Supplementary Information [file 41467_2020_20145_MOESM1_ESM.pdf]

Supplementary Information

Heme biosynthesis depends on previously unrecognized acquisition of iron-sulfur cofactors in human amino-levulinic acid dehydratase

Gang Liu[1], Debangsu Sil[2], Nunziata Maio[1], Wing-Hang Tong[1], J. Martin Bollinger, Jr.[2,3], Carsten Krebs[2,3]* and Tracey Ann Rouault[1]*

[1]*Eunice Kennedy Shriver* National Institute of Child Health and Human Development, National Institutes of Health, Bethesda, MD, USA

[2]Department of Chemistry and [3]Department of Biochemistry and Molecular Biology, The Pennsylvania State University, University Park, PA 16802, USA

*Correspondence: rouault@mail.nih.gov and Krebs- cdk10@psu.edu

Supplementary results and discussion

Impetus to search for more iron-sulfur proteins involved in heme biosynthesis

To attempt to identify as-yet-uncharacterized points of convergence between the Fe-S and heme biosynthetic pathways, we investigated how defective ISC biogenesis, achieved by the knockdown (KD) of the Fe-S assembly scaffold, ISCU, affected heme content in HepG2 cells (Supplementary Figure 2). In cells that lacked ISCU, we overexpressed yeast Fech, which is not an Fe-S protein[1] and is therefore not expected to be affected by the KD of ISCU. We overrode the rate-limiting step of heme biosynthesis catalyzed by ALAS1 by adding 500 μM ALA to the culture medium and used ALAS1 protein levels and the activities of the extra-mitochondrial heme dependent enzymes, CYP1A2 and catalase, as readouts of the integrity of the heme biosynthetic pathway[2, 3, 4]. Upon supplementation with ALA, ALAS1 protein levels were slightly decreased and the activities of catalase and cytochrome P450 1A2 (CYP1A2) were significantly increased (Supplementary Figures 2a-2c, all *P* values <0.01), indicating that heme biosynthesis increased in cells loaded with ALA. As expected, siRNA KD of ISCU impaired Fe-S cluster biogenesis, resulting in the loss of endogenous FECH (Supplementary Figure 2a, lanes 5 and 6), slightly increased ALAS1 protein levels (Supplementary Figure 2a), and significantly reduced CYP1A2 and catalase activities (Supplementary Figures 2b and 2c, all *P* values <0.01). Complementation of the ISCU-depleted cells with the yeast Fech did not restore heme biosynthesis, as gauged by the activities of heme-dependent catalase and CYP1A2 (Supplementary Figures 2a-2c). This negative result is consistent with the possibility that additional intersections existed between the ISC and heme pathways, though it cannot be definitively interpreted because failure to rescue can occur from many causes.

As KD of ISCU is known to affect both the mitochondrial and cytosolic ISC pathways[5], and heme biosynthesis is carried out by enzymes that localize to both compartments[6], we dissected the effect of impaired cytosolic ISC biogenesis on heme biosynthesis. Confined disruption of the solely cytosolic ISC biogenesis was achieved by expressing a mutant of the cytosolic isoform (an isoform from which a mitochondrial targeting sequence was deleted) of the main scaffold ISCU that harbored a substitution of the highly

conserved Asp46 with Ala, ISCU1-D46A (D46A-MYC)[7]. Asp46 of human cytosolic ISCU1 corresponds to Asp71 of mitochondrial isoform ISCU2 and to Asp39 of bacterial IscU. The conserved Asp residue is critical for the initial assembly of nascent Fe-S cofactor[8], and substitution of this residue with Ala was initially found to impair transfer of nascent Fe-S clusters to downstream recipients in bacteria[8, 9]. Later studies found that the Asp to Ala mutations (D46A for ISCU1 and D71A for ISCU2) in mammalian cells also caused Fe-S biosynthesis defects[7, 10]. Upon 24-h induction of the mutagenized cytosolic isoform of ISCU, ISCU1-D46A-MYC, in HEK293T cells treated with ALA to upregulate the heme biosynthetic pathway, FECH protein levels were unaffected, demonstrating that the mitochondrial ISC pathway was intact (Supplementary Figure 2d). Nonetheless, ALAS1 protein levels increased, whereas activities of the non-mitochondrial heme proteins, CYP1A2 and catalase, were markedly reduced as compared with ALA-treated control cells (EV-MYC) (Supplementary Figures 2d-2f, all P values <0.01). These results suggested that heme biosynthesis was suppressed when cytosolic ISC biogenesis was inhibited and that one of the cytosolic heme biosynthetic enzymes was likely an iron-sulfur protein.

Supplementary Table 1 PCR primers used in this study

Primer Name	Sequence (5' to 3')
MBP ALADF	GAAGGATTTCACATATAATGCAGCCCCAGTCCGTT
MBP ALADR	GACGATATCGCGGCCTCATTCCTCCTTCAGCCAC
ALAD-AFR-AAA-F	GCAGCTGCAAGAGCAGGTG
ALAD-AFR-AAA-R	AGTCATGGCCTCCAGTACG
C119A-F	CAGGCAGACGTCAGCGGCCACCAGGAGG
C119A-R	CCTCCTGGTGGCCGCTGACGTCTGCCTG
C122A-F	GTGTAGGGACACAGGGCGACGTCACAGGCCAC
C122A-R	GTGGCCTGTGACGTCGCCCTGTGTCCCTACAC
C124A-F	GGGAGGTGTAGGGAGCCAGGCAGACGTCAC
C124A-R	GTGACGTCTGCCTGGCTCCCTACACCTCCC
C132A-F	CACTCAGGAGCCCGGCGTGACCATGGGAGG
C132A-R	CCTCCCATGGTCACGCCGGGCTCCTGAGTG
C162A-F	GTATGCCAAGGCAGGAGCTCAGGTGGTAGCCC
C162A-R	GGGCTACCACCTGAGCTCCTGCCTTGGCATAC
C223A-F	GGCAGCTGGTAGGCGCGGCGGTCCCC
C223A-R	GGGGACCGCCGCGCCTACCAGCTGCC
K252M-F	CATTCCCGGCATCACCATGAGCATGTCAGC
K252M-R	GCTGACATGCTCATGGTGATGCCGGGAATG

Supplementary figures and legends

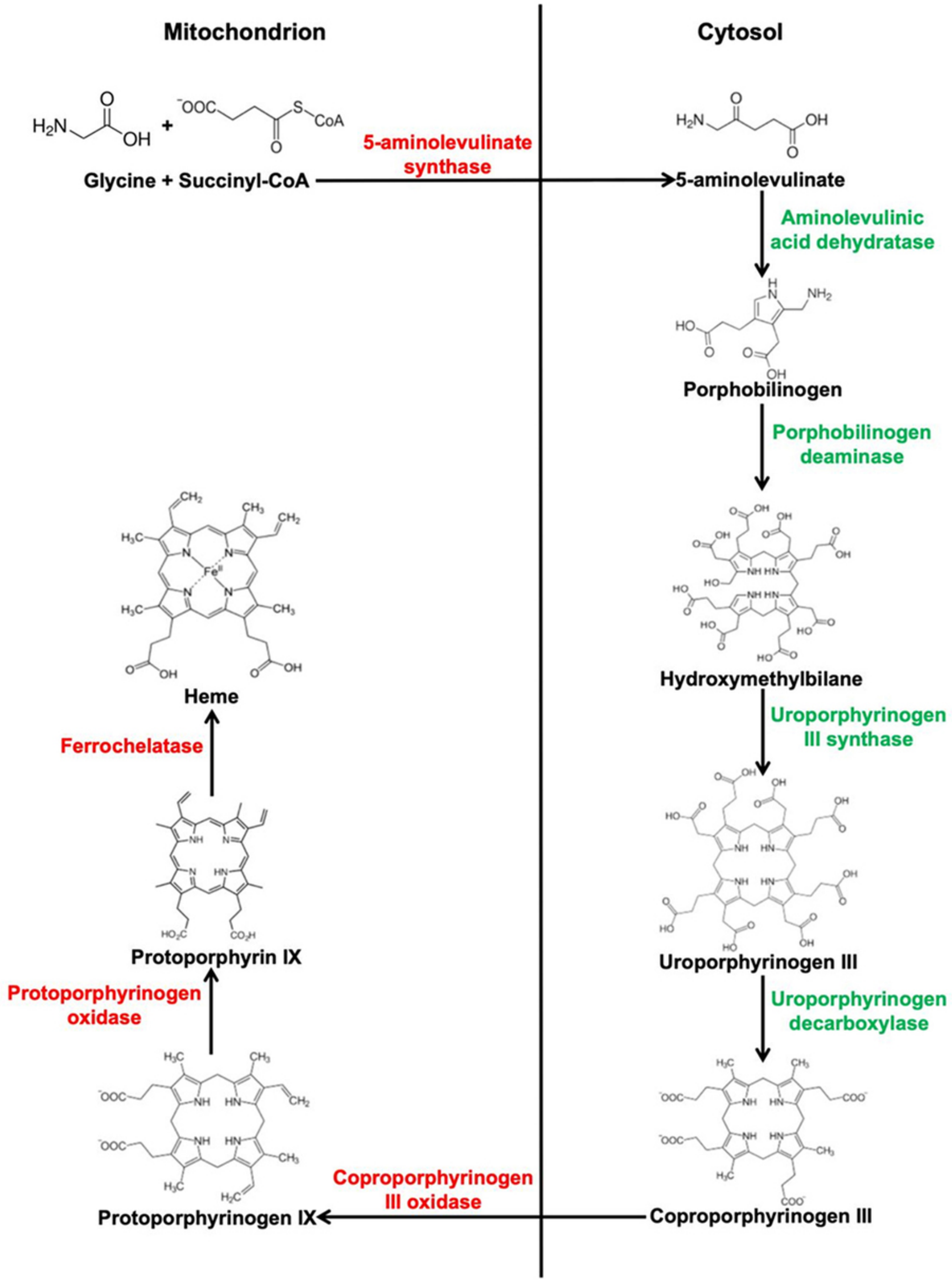

Supplementary Figure 1. A simplified schematic of the heme biosynthesis pathway.

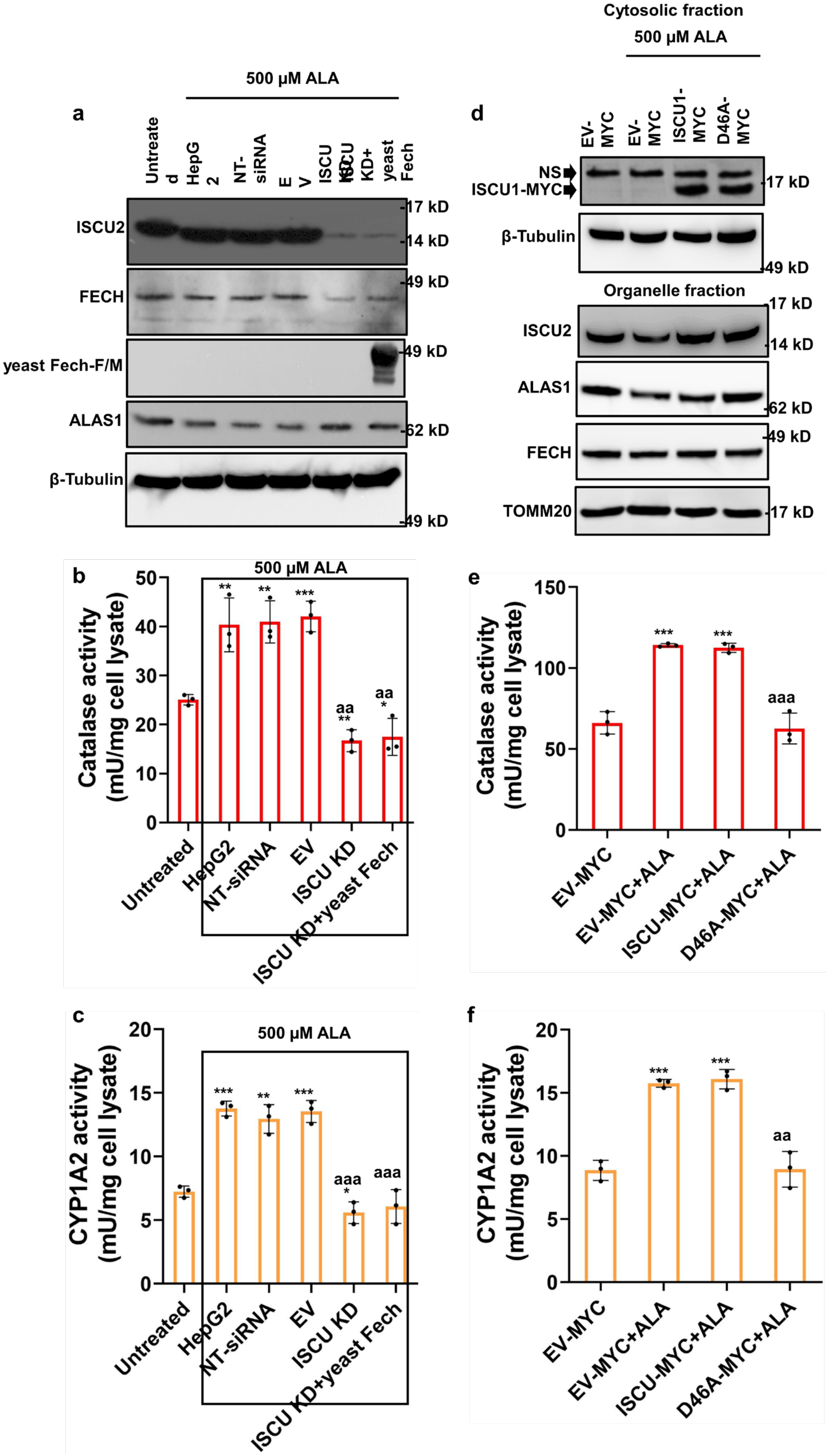

a
500 μM ALA
Untreated
HepG2
NT-siRNA
EV
ISCU KD
ISCU KD+ yeast Fech
ISCU2
FECH
yeast Fech-F/M
ALAS1
β-Tubulin
17 kD
14 kD
49 kD
62 kD
d
Cytosolic fraction
500 μM ALA
EV-MYC
ISCU1-MYC
D46A-MYC
NS
ISCU1-MYC
β-Tubulin
Organelle fraction
ISCU2
ALAS1
FECH
TOMM20
b
Catalase activity (mU/mg cell lysate)
e
EV-MYC+ALA
ISCU-MYC+ALA
D46A-MYC+ALA
c
CYP1A2 activity (mU/mg cell lysate)
f

Supplementary Figure 2. Although Human FECH is a known Fe-S protein, another Fe-S protein is implicated as being involved in heme biosynthesis in non-erythroid cells

(a) Western blot analysis of protein levels of ISCU, FECH, yeast FECH and ALAS1 in untreated HepG2 cells, HepG2 cells treated with 500 μM ALA, or 500 μM ALA treated HepG2 cells transfected with non-targeting siRNA (NT-siRNA), pCMV-Entry empty vector (EV), human ISCU siRNA (ISCU KD) or the combination of human ISCU siRNA+ pCMV6-yeast FECH-FLAG/MYC (ISCU KD+yeast FECH). The protein levels of human β-Tubulin served as a loading control. These experiments were repeated at least twice independently with similar results. **(b)** Catalase and **(c)** CYP1A2 activities in lysates of these cells. **(d)** Western blot analysis of protein levels of cytosolic ISCU1-MYC and ISCU2, ALAS1 and FECH in organellar fractions of untreated HEK293T cells stably transfected with empty vector (EV-MYC, untreated control) and 500 μM ALA treated EV-MYC cells (treated control), HEK293T cells stably expressing wild-type ISCU1 (ISCU1-MYC), and cells stably expressing the ISCU1 D46A mutant (D46A-MYC). Protein levels of β-Tubulin and TOM20 served as loading controls for the cytosolic (left panel) and organelle (right panel) fractions, respectively. NS means non-specific bands. These experiments were repeated twice independently with similar results. **(e)** Catalase and **(f)** CYP1A2 activities in lysates of cells treated as in panel A. The data in panels **(b)**, **(c)**, **(e)** and **(f)** are presented as mean values ± standard deviations of n=3 biologically independent samples. Two-sided unpaired Student's *t* test was employed to analyze statistical significance. No multiple comparison was performed. *, **, *** represent P<0.05, P<0.01 and P<0.001, respectively, versus the "untreated" or the "EV-MYC" group; ^{aa}, ^{aaa} represent P<0.01 and P<0.001, respectively, versus the "HepG2" or the "EV-MYC+ALA" group**.** The exact *P* values are provided in the Source Data file.

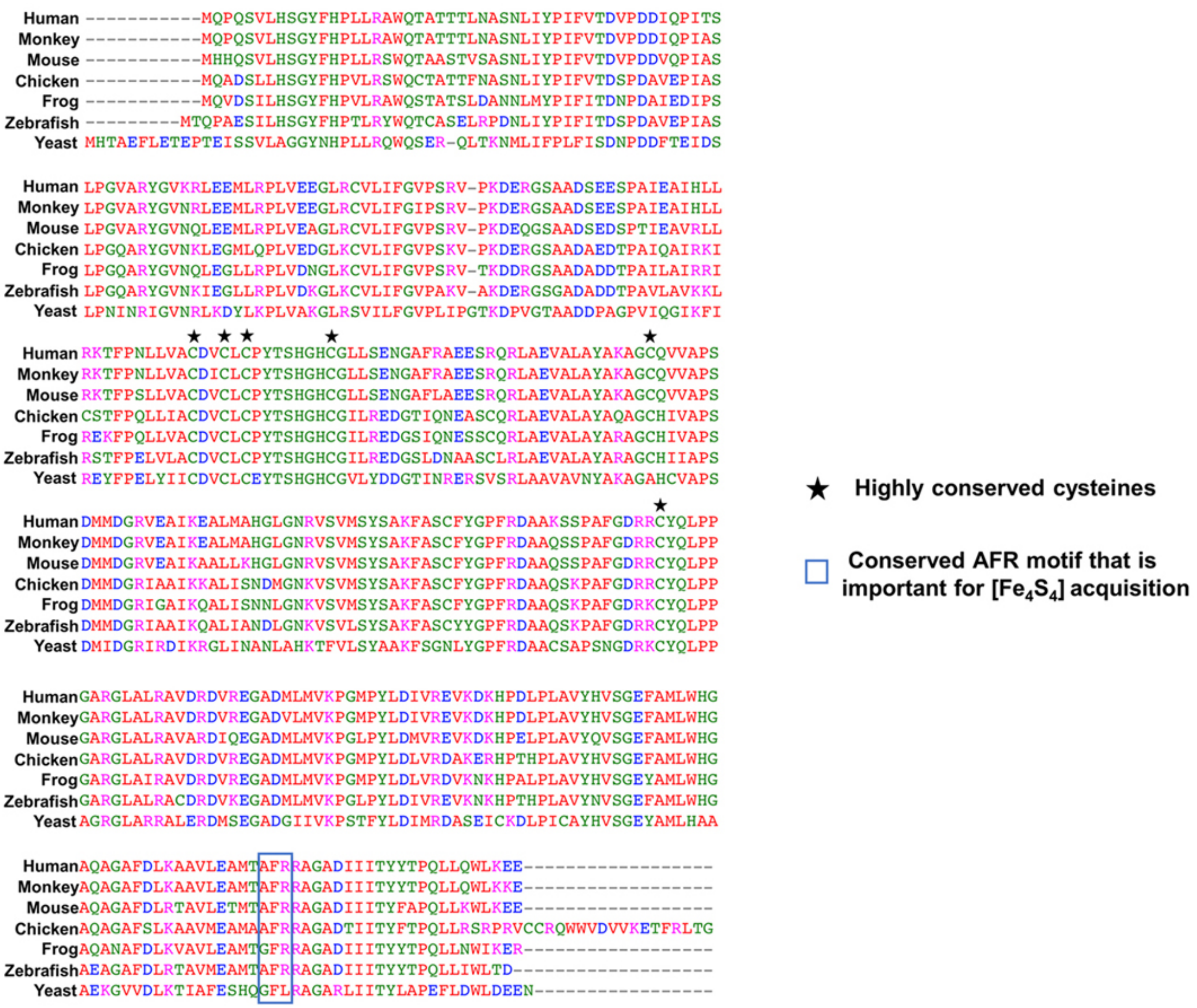

Supplementary Figure 3. Sequence alignment of eukaryotic ALAD orthologs. The alignment shows that the AFR motif and Fe-S ligating cysteines are highly conserved in a broad range of species.

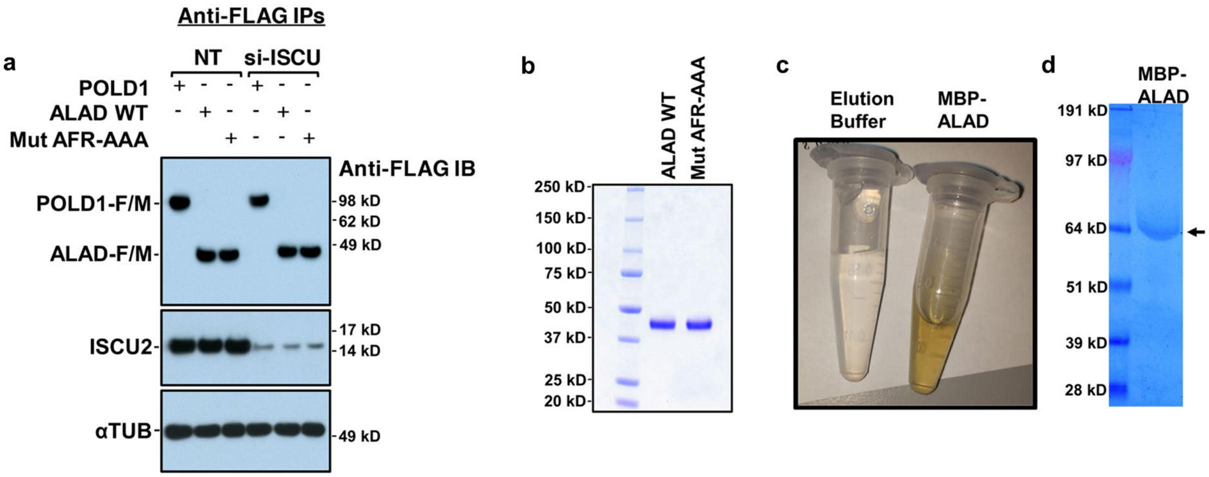

Supplementary Figure 4. Characterization of the color and expression levels of ALAD protein expressed in human cells and bacteria. (a) Immunoblot (IB) analysis of levels of the recombinant proteins subjected to ^{55}Fe incorporation analysis in Figure 1c. **(b)** Coomassie staining analysis of C-terminally FLAG/MYC-tagged ALAD WT and Mut AFR-AAA that were expressed in Expi293 cells and purified anaerobically. **(c)** Photograph of elution buffer (left) and MBP-ALAD expressed along with *isc* operon in *E. coli* and purified anaerobically (right), which showed brown coloration of overexpressed holo-ALAD. **(d)** Coomassie staining of the MBP-ALAD anaerobically purified from *E. coli*. All these experiments were repeated at least twice independently with similar results

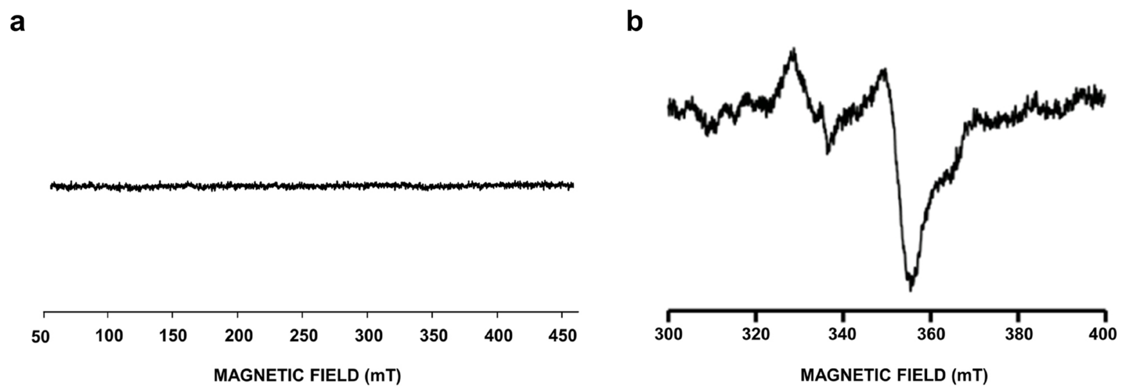

Supplementary Figure 5. EPR spectra of MBP-ALAD samples with or without dithionite reduction. EPR spectra of **(a)** MBP-ALAD expressed along with the *isc* operon in *E. coli* and purified anaerobically showing the absence of any paramagnetic species with half integer spin; and **(b)** MBP-ALAD reduced with $Na_2S_2O_4$ showing a weak signal originating from the $[Fe_4S_4]^+$ cluster.

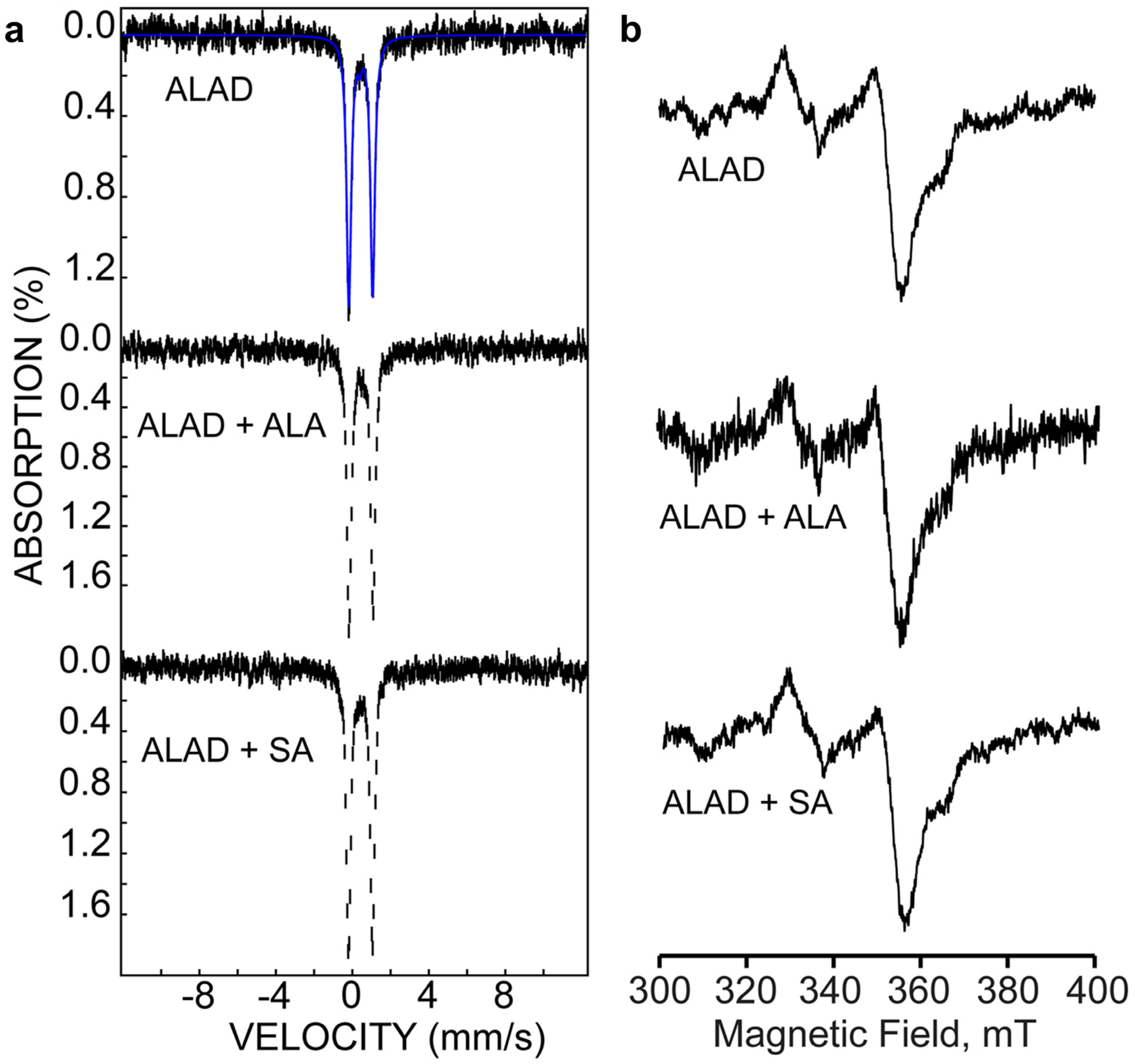

Supplementary Figure 6. Mössbauer and EPR spectra of as purified or dithionite-reduced MBP-ALAD samples added with ALA or SA. (a) 4.2-K/53-mT Mössbauer spectra (black vertical bars) of the anaerobically purified MBP-ALAD (top) in presence of 10 mM ALA (middle) and 10 mM SA (bottom). The blue line represents simulation with parameters quoted in the text. **(b)** X-band EPR spectra of ALAD (150 μM) at 20 K in the presence of 2.5 mM dithionite (top) and 10 mM ALA (middle) or 10 mM SA (bottom).

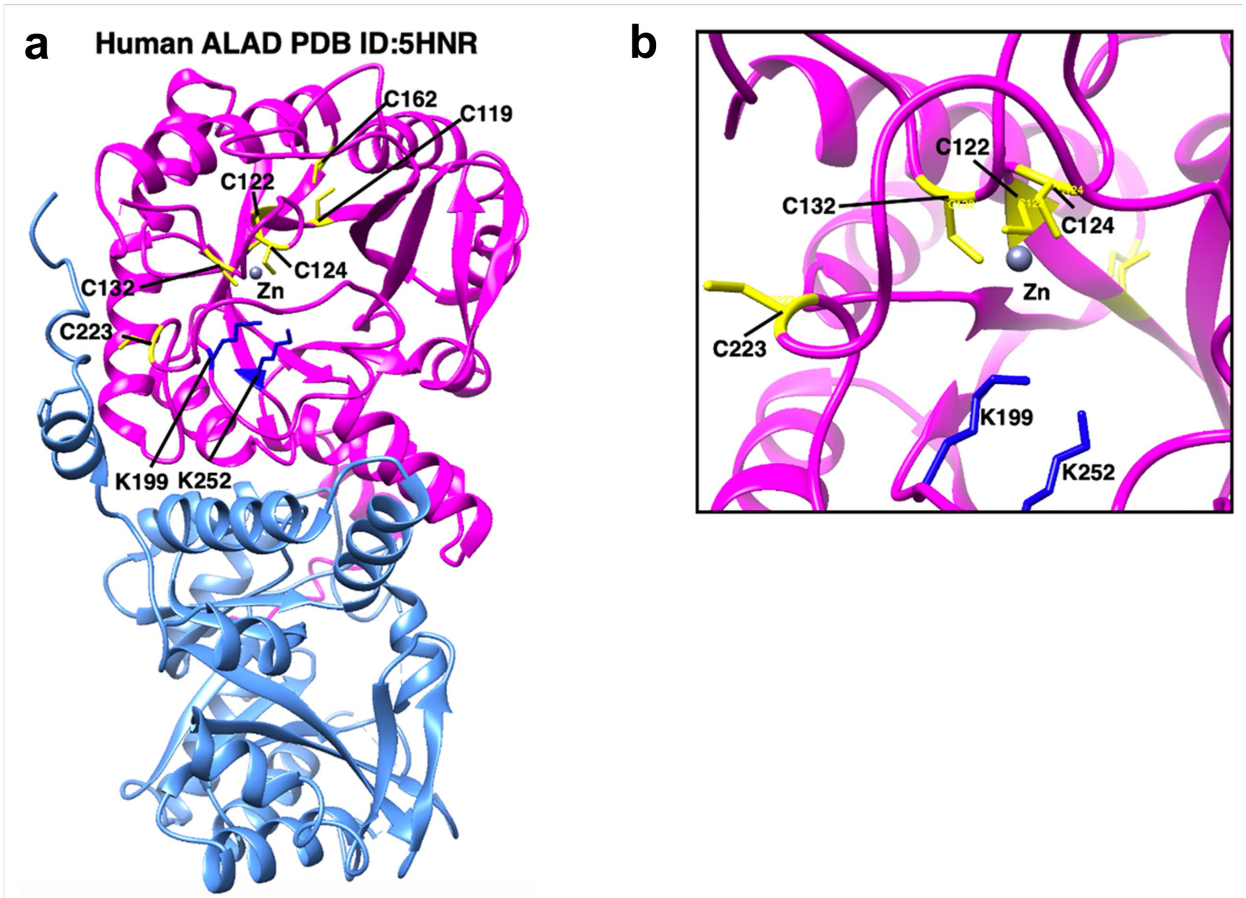

Supplementary Figure 7. Crystal structure of zinc-ALAD (PDB: 5HNR). (a) A dimer of ALAD is shown for simplicity, with one monomer highlighted in magenta and the adjacent in blue. Cysteines 119, 122, 124, 132, 162 and 223 are shown in yellow, while lysine residues 199 and 252 are highlighted in blue. Lysine 252 is known to engage in a Schiff base bond with the substrate ALA, whereas the role of lysine 199 remains unknown. **(b)** The cysteines that ligate zinc in the previously solved structure are C122, C124 and C132. The proximity of C162 to C119 suggests that these two residues may engage in disulfide bond formation, as previously shown[16]. C223 protrudes at the interface between adjacent monomers, likely contributing to ALAD oligomerization by contacting residues on the near protomer. An alternative possibility is that C223 may represent the fourth cysteine residue ligating the Fe-S cluster, given its proximity to C122, C124 and C132.

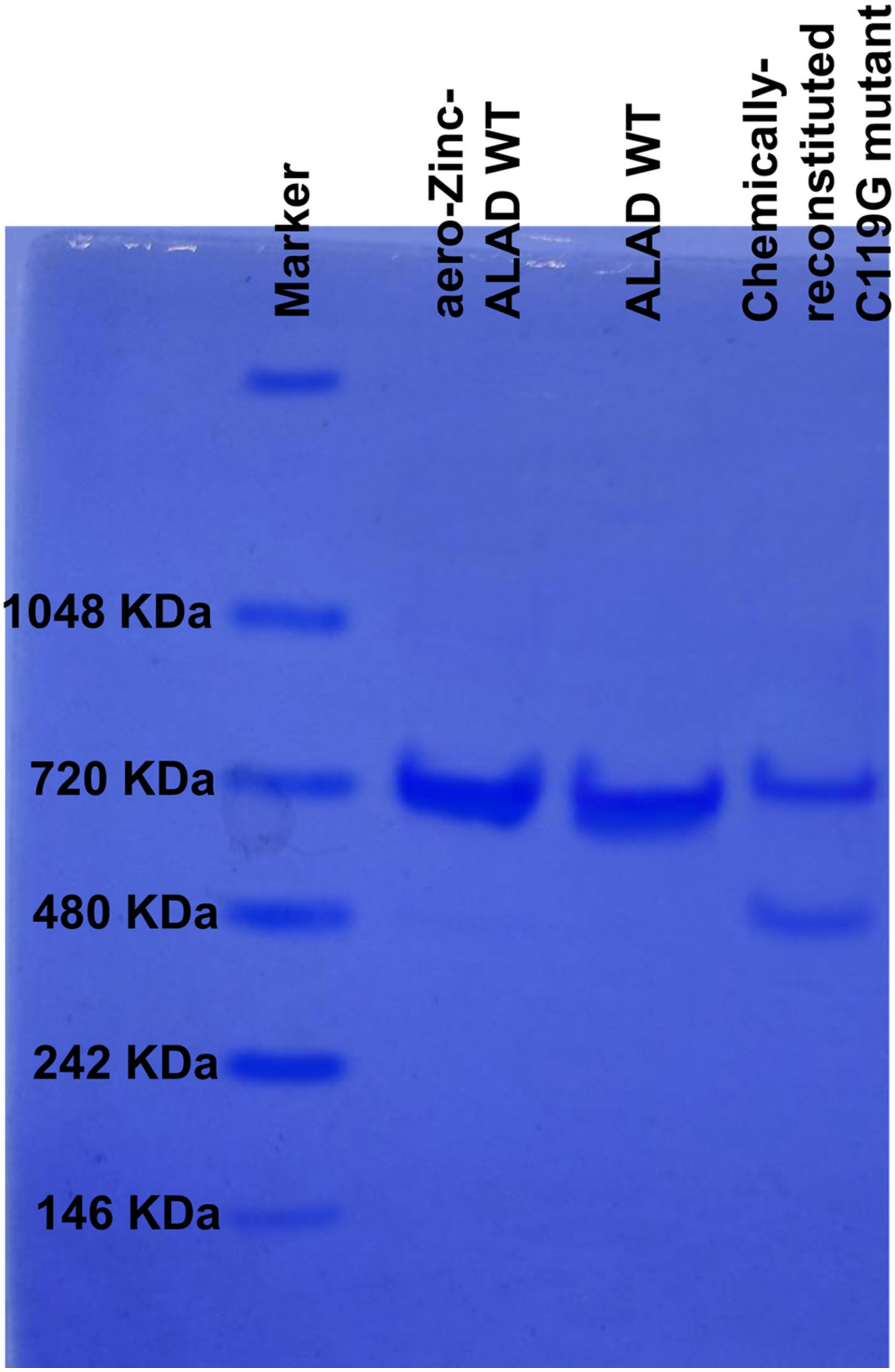

Supplementary Figure 8. The picture of original gel showing the Native PAGE results for aero-Zinc-ALAD WT and ALAD WT. The experiment was repeated twice independently with similar results.

Supplementary references

1. Dailey HA, Finnegan MG, Johnson MK. Human Ferrochelatase Is an Iron-Sulfur Protein. *Biochemistry* **33**, 403-407 (1994).

2. Sinclair PR, Gorman N, Jacobs JM. Measurement of heme concentration. *Curr Protoc Toxicol* **Chapter 8**, Unit 8 3 (2001).

3. Correia MA, Sinclair PR, De Matteis F. Cytochrome P450 regulation: the interplay between its heme and apoprotein moieties in synthesis, assembly, repair, and disposal. *Drug Metab Rev* **43**, 1-26 (2011).

4. Vlasits J, Jakopitsch C, Bernroitner M, Zamocky M, Furtmuller PG, Obinger C. Mechanisms of catalase activity of heme peroxidases. *Arch Biochem Biophys* **500**, 74-81 (2010).

5. Tong WH, Rouault TA. Functions of mitochondrial ISCU and cytosolic ISCU in mammalian iron-sulfur cluster biogenesis and iron homeostasis. *Cell Metab* **3**, 199-210 (2006).

6. Ponka P. Tissue-specific regulation of iron metabolism and heme synthesis: distinct control mechanisms in erythroid cells. *Blood* **89**, 1-25 (1997).

7. Kim KS, Maio N, Singh A, Rouault TA. Cytosolic HSC20 integrates de novo iron-sulfur cluster biogenesis with the CIAO1-mediated transfer to recipients. *Hum Mol Genet* **27**, 837-852 (2018).

8. Marinoni EN*, et al.* (IscS-IscU)2 Complex Structures Provide Insights into Fe2S2 Biogenesis and Transfer. *Angew Chem Int Ed* **51**, 5439-5442 (2012).

9. Raulfs EC, O'Carroll IP, Dos Santos PC, Unciuleac MC, Dean DR. In vivo iron-sulfur cluster formation. *Proc Natl Acad Sci U S A* **105**, 8591-8596 (2008).

10. Crooks DR*, et al.* Acute loss of iron-sulfur clusters results in metabolic reprogramming and generation of lipid droplets in mammalian cells. *J Biol Chem* **293**, 8297-8311 (2018).
