# Supplementary material for: Heme biosynthesis depends on previously unrecognized acquisition of iron-sulfur cofactors in human amino-levulinic acid dehydratase
Source: Nat Commun. 2020 Dec 9;11:6310. doi: 10.1038/s41467-020-20145-9 (PMC7725820; doi:10.1038/s41467-020-20145-9)
Supplement: Supplementary file 3 — Reporting Summary [file 41467_2020_20145_MOESM3_ESM.pdf]

nature research

Corresponding author(s): Tracey Rouault

Last updated by author(s): Nov 2, 2020

Reporting Summary

Nature Research wishes to improve the reproducibility of the work that we publish. This form provides structure for consistency and transparency in reporting. For further information on Nature Research policies, see Authors & Referees and the Editorial Policy Checklist .

Statistics

For all statistical analyses, confirm that the following items are present in the figure legend, table legend, main text, or Methods section.

n/a	Confirmed	
☐	☒	The exact sample size (*n*) for each experimental group/condition, given as a discrete number and unit of measurement
☐	☒	A statement on whether measurements were taken from distinct samples or whether the same sample was measured repeatedly
☐	☒	The statistical test(s) used AND whether they are one- or two-sided<br>*Only common tests should be described solely by name; describe more complex techniques in the Methods section.*
☒	☐	A description of all covariates tested
☒	☐	A description of any assumptions or corrections, such as tests of normality and adjustment for multiple comparisons
☐	☒	A full description of the statistical parameters including central tendency (e.g. means) or other basic estimates (e.g. regression coefficient) AND variation (e.g. standard deviation) or associated estimates of uncertainty (e.g. confidence intervals)
☐	☒	For null hypothesis testing, the test statistic (e.g. *F*, *t*, *r*) with confidence intervals, effect sizes, degrees of freedom and *P* value noted<br>*Give P values as exact values whenever suitable.*
☒	☐	For Bayesian analysis, information on the choice of priors and Markov chain Monte Carlo settings
☒	☐	For hierarchical and complex designs, identification of the appropriate level for tests and full reporting of outcomes
☒	☐	Estimates of effect sizes (e.g. Cohen's *d*, Pearson's *r*), indicating how they were calculated

Our web collection on statistics for biologists contains articles on many of the points above.

Software and code

Policy information about availability of computer code

Data collection	Image J v1.46 was used to analyze band intensity of Western blotting.
Data analysis	GraphPad Prism 8 was used to calculate the P values. Microsoft Excel 2016 was used for calculating means and standard deviations of the data. Statistical analyses of 55Fe labeling experiments were performed with GraphPad Prism 8.

For manuscripts utilizing custom algorithms or software that are central to the research but not yet described in published literature, software must be made available to editors/reviewers. We strongly encourage code deposition in a community repository (e.g. GitHub). See the Nature Research guidelines for submitting code & software for further information.

Data

Policy information about availability of data

All manuscripts must include a data availability statement. This statement should provide the following information, where applicable:

- Accession codes, unique identifiers, or web links for publicly available datasets
- A list of figures that have associated raw data
- A description of any restrictions on data availability

All the data reported in this study are available upon reasonable request. Databases used in this study include NCBI (https://www.ncbi.nlm.nih.gov/), PDB (https://www.rcsb.org/), and UniProt (https://www.uniprot.org/). Source data are also provided with this paper.

Field-specific reporting

Please select the one below that is the best fit for your research. If you are not sure, read the appropriate sections before making your selection.

Life sciences study design

All studies must disclose on these points even when the disclosure is negative.

Sample size	Three independent replicates were performed for most of the experiments (for 55Fe incorporation experiments we performed four replicates). No sample-size calculation was performed. Three replicates were sufficient to show significant differences between different experimental groups.
Data exclusions	No data exclusion.
Replication	All experiments were successfully replicated with similar results for at least two times.
Randomization	Not relevant. This study doesn't include grouping.
Blinding	Investigators were not blinded to information of cell lines and protein samples because the information was important for choosing appropriate kits, antibodies and analysis methods

Reporting for specific materials, systems and methods

We require information from authors about some types of materials, experimental systems and methods used in many studies. Here, indicate whether each material, system or method listed is relevant to your study. If you are not sure if a list item applies to your research, read the appropriate section before selecting a response.

Materials & experimental systems

n/a	Involved in the study	
☐	☒	Antibodies
☐	☒	Eukaryotic cell lines
☒	☐	Palaeontology
☒	☐	Animals and other organisms
☒	☐	Human research participants
☒	☐	Clinical data

Methods

n/a	Involved in the study	
☒	☐	ChIP-seq
☒	☐	Flow cytometry
☒	☐	MRI-based neuroimaging

Antibodies

Antibodies used	rabbit anti-ALAD (1:1000), Abcam ab59013; rabbit anti-ALAS1 (1:500), Abcam ab84962; rabbit anti-FECH (1:500), Abcam ab137042; mouse anti-TOMM20 (1:2000), Abcam ab56783; mouse anti-β-Tubulin (1:2000), Sigma T8328; mouse anti-α-Tubulin (1:2000), Sigma T5168; rabbit anti-HSC20 (1:50 for Co-IP experiments), Sigma HPA018447; mouse anti-HSC20 (1:1000), Origene TA507285; mouse anti-FLAG Tag (1:100 for Co-IP experiments), Genscript A00187; rabbit anti-MT2A (1:200), LifeSpan Bio LS-C667906-50; mouse anti-FLAG (1:2000), OriGene TA50011-100; rabbit anti-ISCU antibody (1;1000) was prepared by our own group. Normal rabbit IgG (not an antibody, served as a negative control for IP experiments), Santa Cruz sc-2345.
Validation	The rabbit anti-ISCU antibody was validated for use for Western blotting analysis of protein samples obtained from human cells in our previous study by Tong et al. (https://www.sciencedirect.com/science/article/pii/S1550413106000635). In the paper, the authors successfully detected both cytosolic and mitochondrial ISCUs in HeLa cells using this antibody.<br>All the other antibodies were validated by the manufacturers for use for Western blotting/immunoprecipitation analysis of protein samples obtained from human cells. rabbit anti-ALAD (https://www.abcam.com/alad-antibody-ab59013.html); rabbit anti-ALAS1 (https://www.abcam.com/alas1-antibody-mitochondrial-marker-ab84962.html); rabbit anti-FECH (https://www.abcam.com/fech-antibody-epr8312-ab137042.html); mouse anti-TOMM20 (https://www.abcam.com/tomm20-antibody-mitochondrial-marker-ab56783.html); mouse anti-β-Tubulin (https://www.sigmaaldrich.com/content/dam/sigma-aldrich/docs/Sigma/Datasheet/2/t8328dat.pdf); mouse anti-α-Tubulin (https://www.sigmaaldrich.com/catalog/product/sigma/t5168?lang=en&region=US); rabbit anti-HSC20 (https://www.sigmaaldrich.com/catalog/product/sigma/hpa018447?lang=en&region=US); mouse anti-HSC20 (https://www.thermofisher.com/antibody/product/HSCB-Antibody-clone-OTI2B7-Monoclonal/TA507285); mouse anti-FLAG Tag (https://www.genscript.com/antibody/A00187-_THE_sup_TM_sup_DYKDDDDK_Tag_Antibody_mAb_Mouse.html); rabbit anti-MT2A (https://www.lsbio.com/antibodies/mt2a-antibody-metallothionein-2a-antibody-ihc-wb-western-ls-c667906/682356); mouse anti-FLAG (https://www.origene.com/catalog/antibodies/tag-antibodies/ta50011-100/clone-oti4c5-anti-ddk-flag-monoclonal-antibody)

Eukaryotic cell lines

Policy information about cell lines

Cell line source(s)	HepG2, ATCC (ATCC® HB-8065™); HeLa, ATCC (ATCC® CCL-2™); HEK293, ATCC (ATCC® CRL-1573™); Expi293F® Cells, ThermoFisher Scientific (A14635); stably-transfected HEK293T cell lines were established using HEK293T cells obtained from ATCC (ATCC® CRL-3216™) and successfully used for investigating the functions of wild-type and mutant ISCUs in our previous studies (https://academic.oup.com/hmg/article/27/5/837/4788600).

Authentication	The HEK293T stable cell lines were authenticated by our previous study using morphology and PCR-based approaches. The HepG2, HeLa, HEK293 and Expi293F® cell lines were directly obtained from and authenticated by ATCC or ThermoFisher Scientific using morphology, karyotyping, and PCR-based approaches.
Mycoplasma contamination	We confirm that all the cell lines used in this study DO NOT have mycoplasma contamination.
Commonly misidentified lines (See ICLAC register)	We used stably-transfected HEK293T cell lines generated by our previous study because they are ready to use. We used HEK293 and Expi293F® Cells because they are suitable for overexpressing target protein.
